# Downregulation of the endothelial histone demethylase JMJD3 is associated with neointimal hyperplasia of arteriovenous fistulas in kidney failure

**DOI:** 10.1016/j.jbc.2022.101816

**Published:** 2022-03-10

**Authors:** Shaozhen Feng, Eric K. Peden, Qunying Guo, Tae Hoon Lee, Qingtian Li, Yuhui Yuan, Changyi Chen, Fengzhang Huang, Jizhong Cheng

**Affiliations:** 1 Department of Nephrology, The First Affiliated Hospital, Sun Yat-Sen University, and Guangdong Provincial Key Laboratory of Nephrology, Guangzhou, China; 2 Selzman Institute for Kidney Health, Section of Nephrology, Department of Medicine, Baylor College of Medicine, Houston, USA; 3 Department of Vascular Surgery, DeBakey Heart and Vascular Institute, Houston Methodist Hospital, Houston, USA; 4 Department of Surgery, Department of Medicine, Baylor College of Medicine, Houston, Texas, USA

**Keywords:** JMJD3, H3K27me3, endothelial cells, neointimal hyperplasia, arteriovenous fistula, α-SMA, α-smooth muscle actin, AVF, arteriovenous fistula, BUN, blood urea nitrogen, ChIP–PCR, chromatin immunoprecipitation–PCR, CKD, chronic kidney disease, DMEM, Dulbecco's Modified Eagle's Medium, EC, endothelial cell, EndMT, endothelial–mesenchymal transition, eNOS, endothelial nitric oxide synthase, FBS, fetal bovine serum, FSP-1, fibroblast-specific protein 1, H3K27, histone H3 lysine 27, JMJD3, Jumonji domain–containing protein-3, L-NAME, *N*(omega)-nitro-l-arginine methyl ester, MTS, 3-(4,5-dimethylthiazol-2-yl)-5-(3-carboxymethoxyphenyl)-2-(4-sulfophenyl)-2H-tetrazolium, NIH, neointimal hyperplasia, NO, nitric oxide, PCNA, proliferating cell nuclear antigen, sgRNA, single-guide RNA, SNP, sodium nitroprusside, TGFβ1, transforming growth factor beta 1, VE-cadherin, vascular endothelial-cadherin, VSMC, vascular smooth muscle cell

## Abstract

Jumonji domain–containing protein-3 (JMJD3), a histone H3 lysine 27 (H3K27) demethylase, promotes endothelial regeneration, but its function in neointimal hyperplasia (NIH) of arteriovenous fistulas (AVFs) has not been explored. In this study, we examined the contribution of endothelial JMJD3 to NIH of AVFs and the mechanisms underlying JMJD3 expression during kidney failure. We found that endothelial JMJD3 expression was negatively associated with NIH of AVFs in patients with kidney failure. JMJD3 expression in endothelial cells (ECs) was also downregulated in the vasculature of chronic kidney disease (CKD) mice. In addition, specific knockout of endothelial JMJD3 delayed EC regeneration, enhanced endothelial mesenchymal transition, impaired endothelial barrier function as determined by increased Evans blue staining and inflammatory cell infiltration, and accelerated neointima formation in AVFs created by venous end to arterial side anastomosis in CKD mice. Mechanistically, JMJD3 expression was downregulated *via* binding of transforming growth factor beta 1–mediated Hes family transcription factor Hes1 to its gene promoter. Knockdown of JMJD3 enhanced H3K27 methylation, thereby inhibiting transcriptional activity at promoters of EC markers and reducing migration and proliferation of ECs. Furthermore, knockdown of endothelial JMJD3 decreased endothelial nitric oxide synthase expression and nitric oxide production, leading to the proliferation of vascular smooth muscle cells. In conclusion, we demonstrate that decreased expression of endothelial JMJD3 impairs EC regeneration and function and accelerates neointima formation in AVFs. We propose increasing the expression of endothelial JMJD3 could represent a new strategy for preventing endothelial dysfunction, attenuating NIH, and improving AVF patency in patients with kidney disease.

Progressive loss of kidney function to the stage requiring frequent dialysis is a major complication of chronic kidney disease (CKD). More than 1.5 million patients with end-stage renal disease require hemodialysis (1), and this population will continue to grow. Arteriovenous fistula (AVF) is a prevalent vascular access for hemodialysis patients. However, the primary patency rates of AVF range from 50% to 87% at 1 year and are about 40% at 5 years (2, 3, 4). Currently, there remains no effective therapies to prevent AVF maturation failure (5, 6), and the mechanisms for fistula loss are still not clearly understood. Neointimal hyperplasia (NIH) is present in most native veins and AVFs, which is prominent in many patients with kidney failure (7). After AVF creation, expansion of neointima may aggravate inward remodeling leading to the development of stenosis and access failure. Some observations from patients with kidney failure reinforce the assumption that a thicker neointima is responsible for the development of stenosis in AVF and access failure (8, 9, 10, 11).

Epigenetics regulates gene expression and vascular cell function. It plays a variety of roles in vascular smooth muscle cell (VSMC) proliferation and neointima formation after vascular injury (12, 13). Previous studies have shown that CKD complications, such as increased levels of uremic toxins, oxidative stress, and inflammation, can induce epigenetic modifications (14, 15). In addition, disorders of epigenetic regulation increase the risk of developing cardiovascular diseases in CKD patients (16, 17). Recent advances in the biology of vascular reconstruction indicate that dysfunction of endothelial cells (ECs) plays a central role in the development of NIH (18, 19, 20, 21). Endothelial dysfunction has been shown to occur in the peripheral vasculature of patients with both severe and moderate CKD (22, 23). While epigenetic regulators that mediate endothelial dysfunction in NIH of AVF are not clear.

Jumonji domain–containing protein-3 (JMJD3/KDM6b) is a histone H3 lysine 27 (H3K27) demethylase and epigenetically activates gene expression by demethylating histone H3 dimethyl and trimethyl lysine 27 (H3K27me2/3) (24). When H3K27 is trimethylated, it is typically associated with silencing of gene promoters (25). Since JMJD3 plays an important role in the epigenetic regulation of gene expression, it has been reported to affect several cellular processes, including cell differentiation (26), cell proliferation, and migration (27), and senescence (28). JMJD3 could be regulated at the transcriptional level and was upregulated in response to diverse stimuli, such as growth factors and stress signals (29, 30). It also can be downregulated in various diseases, especially cancer and acute myeloid leukemia (31, 32, 33). However, the role of JMJD3 in ECs during NIH development in AVFs and the mechanism of its expression regulation in CKD remain unknown.

Herein, we investigate the association of endothelial JMJD3 with NIH of AVFs in patients with kidney failure and in mouse AVF models created by venous end to arterial side anastomosis configuration with chronic renal failure. We also explore the regulatory mechanism of endothelial JMJD3 expression in CKD.

## Results

### Decreased expression of endothelial JMJD3 is associated with NIH of AVFs from kidney failure patients

**Figure 1:**
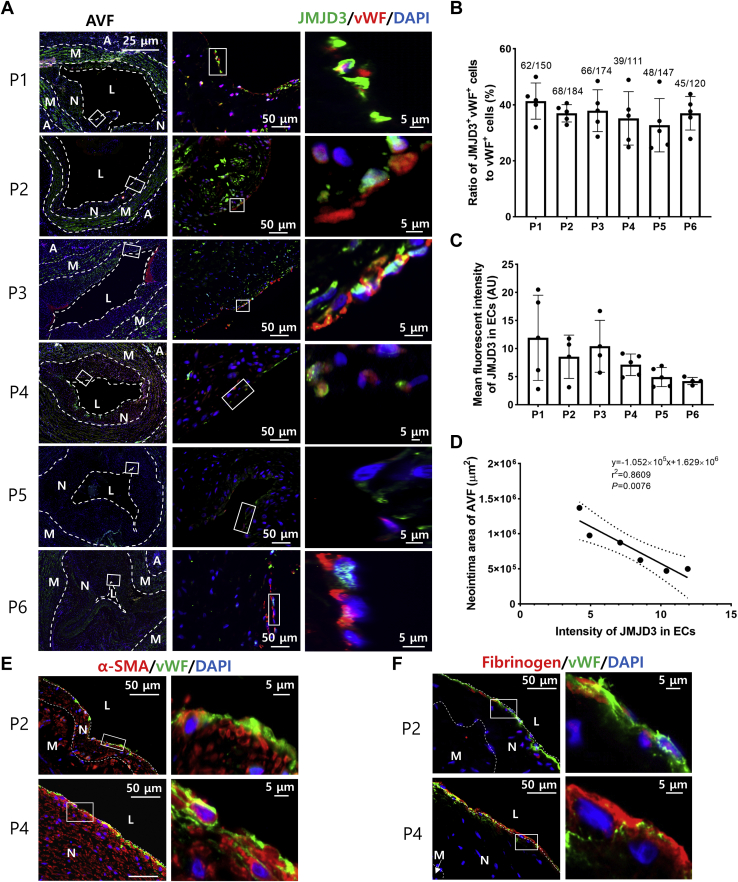
**Epigenetic signatures of AVF in patients with kidney failure.***A*, immunofluorescent staining of JMJD3 (*green*) and vWF (*red*) in venous anastomosis of human AVF. Neointima area and lumen are circumscribed by *dotted lines*. Samples were selected from those specimens having a complete circumference. *B*, percentage of JMJD3^+^/vWF^+^ double-positive cells number in total ECs (vWF^+^) was calculated from five visual fields (40×) of each sample. Data were shown as means ± SD. *C*, density analysis of fluorescence intensity of JMJD3 signals in vWF+ cells from five visual fields (40×) of each sample. Mean of immunofluorescence intensity of JMJD3 was quantified by ImageJ software and expressed as arbitrary unit (AU), which was calculated as (mean pixel intensity × endothelial area)/100,000. Data were shown as mean ± SD. *D*, correlation of fluorescence intensity for endothelial JMJD3 and neointima area of human AVFs (n = 6). Association between two variables was analyzed by Pearson's correlation. *E* and *F*, immunofluorescent staining of α-SMA (*red*), fibrinogen (*red*), and vWF (*green*) in venous anastomosis of human AVFs. Areas of neointima and lumen are circumscribed by *dotted lines*. Photos are representative of six human samples. α-SMA, α-smooth muscle actin; A, adventitia; AVF, arteriovenous fistula; EC, endothelial cell; JMJD3, Jumonji domain–containing protein-3; L, lumen; M, media; N, neointima; P1–6, patients 1 to 6; vWF, von Willebrand factor.

To confirm the role of epigenetic regulation in NIH of AVFs, we examined histone modifications in NIH of AVFs from kidney failure patients. We found that JMJD3 was expressed in the nuclei of ECs (von Willebrand factor plus), and the expression of JMJD3 in the endothelium of venous anastomosis of AVF was reversely correlated with the size of neointima area of AVFs (*p* < 0.05, Fig. 1, *A*–*D*). The lesser expression level of JMJD3 in ECs, the larger area of NIH observed. Furthermore, lower expression of endothelial JMJD3 was accompanied by higher expression of mesenchymal marker α-smooth muscle actin (α-SMA) and endothelial injury component fibrinogen. Positive staining of α-SMA and fibrinogen can be found in the endothelium of venous anastomosis of AVF with varying degrees (Fig. 1, *E* and *F*), which indicate endothelial–mesenchymal transition (EndMT) and endothelium injury. Therefore, these results suggested that decreased JMJD3 expression in the endothelium could be associated with NIH in AVFs probably *via* regulating endothelial dysfunction.

### KO of JMJD3 in ECs promotes NIH of AVFs in CKD mice

**Figure 2:**
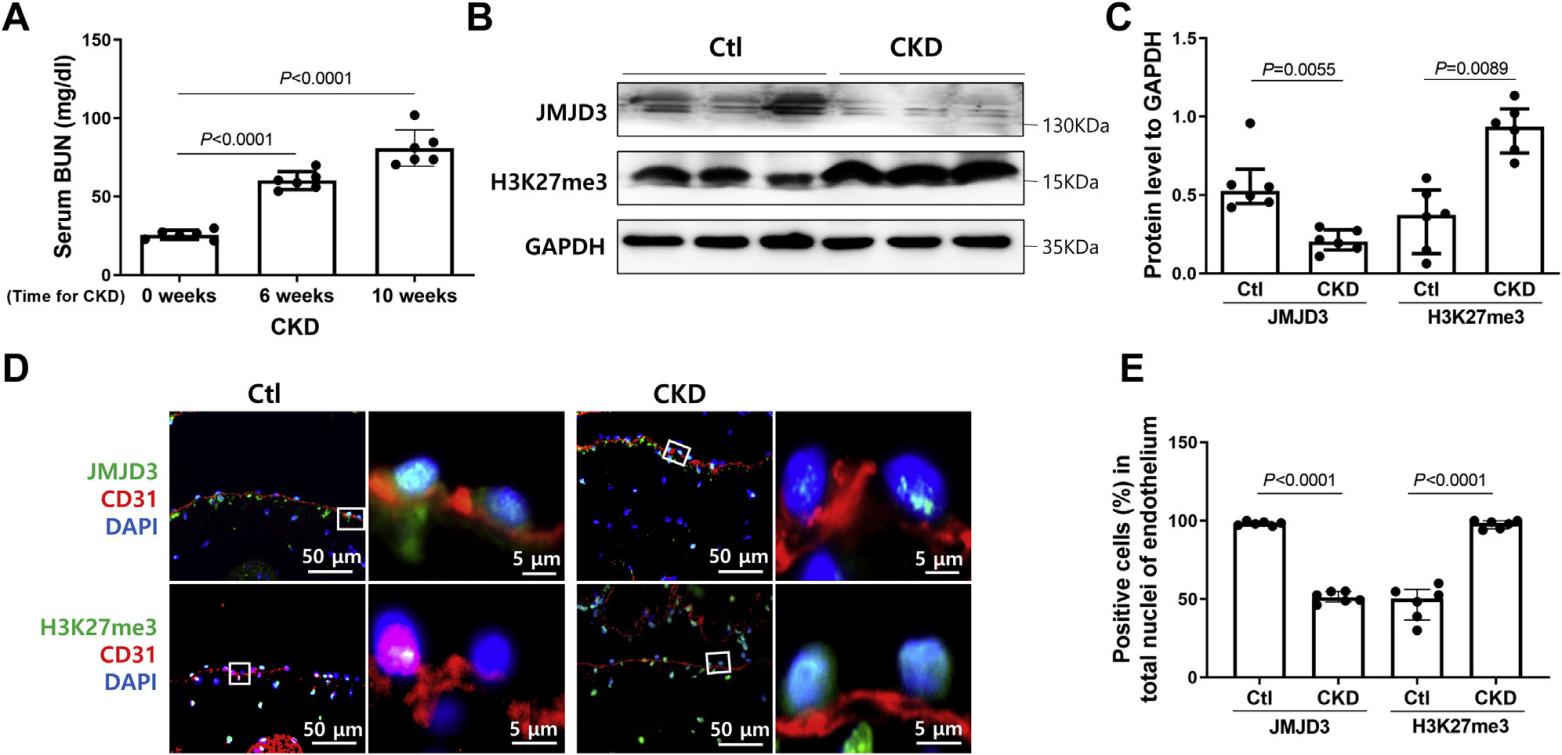
**Expression of endothelial JMJD3 was decreased in vessels of CKD mice.***A*, serum BUN levels were detected in CKD mice before and after operation (n = 6). Data are presented as means ± SD. Statistical significance was measured using one-way ANOVA. *B* and *C*, Western blotting of JMJD3 and H3K27me3 expression in aortas from control (Ctl, n = 6) and CKD mice (n = 6). Quantification analysis of Western blotting shown as means ± SD. *D* and *E*, immunofluorescent staining of JMJD3 (*green*) or H3K27me3 (*green*) and CD31 (*red*) in the external jugular veins of control (Ctl, n = 6) and CKD mice (n = 6). Percentage of JMJD3 or H3K27me3-positive cells in total nuclei of endothelium from each sample was calculated (*E*). BUN, blood urea nitrogen; CKD, chronic kidney disease; H3K27, histone H3 lysine 27; JMJD3, Jumonji domain–containing protein-3.

**Figure 3:**
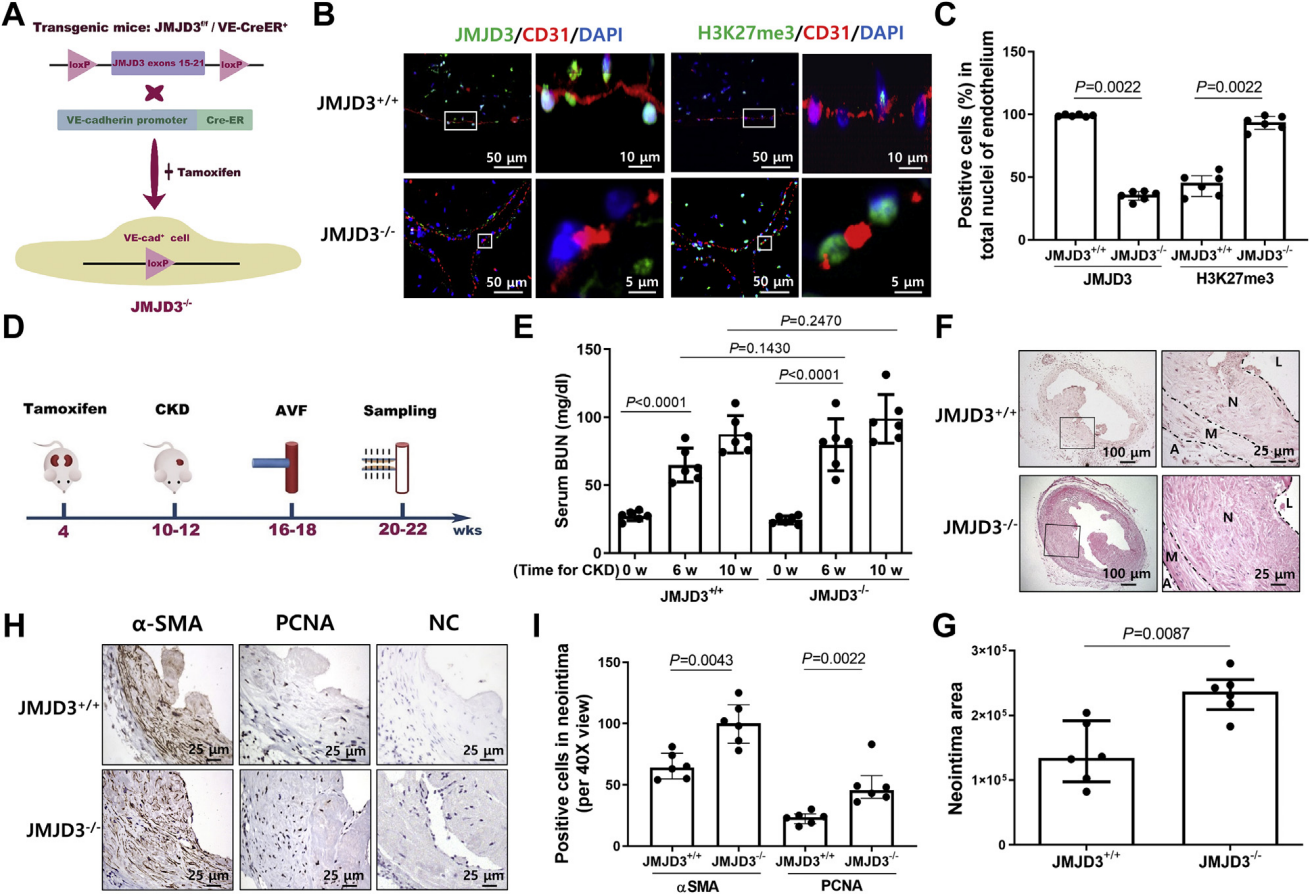
**JMJD3 KO in ECs accelerates neointima formation.***A*, schematic for generating EC^JMJD3 KO^ mice. *B*, EC^JMJD3 KO^ and EC^JMJD3+/+^ were treated with tamoxifen. Immunofluorescent staining of JMJD3 (*green*) or H3K27me3 (*green*) and CD31 (*red*) in the external jugular veins of EC^JMJD3+/+^ and EC^JMJD3 KO^ mice (n = 6). *C*, percentage of JMJD3 or H3K27me3-positive cells in total nuclei of endothelium from each sample was calculated. *D*, schematic for CKD-AVF mice model. CKD and AVFs were performed in the two groups of mice (EC^JMJD3+/+^ and EC^JMJD3 KO^). Sera were collected at the day before surgery of CKD, 6 weeks postsurgery of CKD and 4 weeks after creating AVF. AVFs were collected after 4 weeks of AVF surgery. *E*, serum BUN levels in EC^JMJD3+/+^ and EC^JMJD3 KO^ CKD mice (n = 6) were measured. Data were presented as mean ± SD. Statistical significance was measured using two-way ANOVA. *F* and *G*, H&E staining of venous anastomosis of AVFs from EC^JMJD3+/+^ or EC^JMJD3 KO^ CKD mice (n = 6). The *black dotted line* represents the boundary between neointima (N), lumen (L), media (M), and adventitia (A). Areas of neointima area of AVFs were calculated (*G*). *H*, immunostaining of α-SMA and PCNA in venous anastomosis of AVFs from EC^JMJD3+/+^ or EC^JMJD3 KO^ (n = 6). Sections incubated with anti-rabbit secondary antibody but without primary antibody were used as negative control (NC). *I*, total numbers of α-SMA^+^ or PCNA^+^ cells in neointima area were calculated from five visual fields (40×) of each sample. All statistical analyses between groups were performed by Mann–Whitney test. Data were shown as means ± SD. α-SMA, α-smooth muscle actin; AVF, arteriovenous fistula; BUN, blood urea nitrogen; CKD, chronic kidney disease; EC, endothelial cell; H3K27, histone H3 lysine 27; JMJD3, Jumonji domain–containing protein-3; PCNA, proliferating cell nuclear antigen.

To assess whether JMJD3 expression in ECs was regulated by uremia, we measured the expression of JMJD3 in blood vessel in normal control and CKD mice. The serum level of blood urea nitrogen (BUN) was remarkably increased from 6 weeks after subtotal nephrectomy in mice, which indicated that the CKD model was successfully established (Fig. 2*A*). JMJD3 expression was decreased significantly in vessels of CKD mice, especially in ECs (CD31^+^) of vein and aortas accompanied by increased H3K27me3 level (Fig. 2, *B*–*E* and S1). To examine the role of endothelial JMJD3 in vascular remodeling of AVF in CKD, JMJD3 was conditionally knocked out in ECs by breeding JMJD3^f/f^ mice with vascular endothelial-cadherin (VE-cadherin)-CreER^T2^ mice (Fig. 3*A*). As expected, KO of JMJD3 in ECs led to a significant increase in H3K27me3 expression (Fig. 3, *B* and *C*). Subtotal nephrectomy and AVF were then created in EC^JMJD3+/+^ and EC^JMJD3 KO^ mice (Fig. 3*D*). Though the serum BUN level was significantly higher in mice after subtotal nephrectomy, there was no statistically significant difference of BUN levels between EC^JMJD3 KO^ mice and EC^JMJD3+/+^ mice observed either at 6 weeks or 10 weeks after CKD surgery, indicating that endothelial KO of JMJD3 did not aggravate renal dysfunction in mice (Fig. 3*E*). In AVFs, JMJD3 KO in ECs dramatically increased NIH and the ratio of neointima to lumen when compared with that in EC^JMJD3+/+^ mice (Fig. 3, *F* and *G*). There were more α-SMA^+^ and proliferating cell nuclear antigen-positive (PCNA^+^) cells in AVFs created in EC^JMJD3 KO^ mice *versus* that in EC^JMJD3+/+^ mice (Fig. 3, *H* and *I*). Therefore, these findings indicated that JMJD3 KO in ECs could promote NIH and VSMC proliferation of AVF.

### JMJD3 deficiency in ECs associates with decreased EC regeneration, increased EndMT, inflammation, and fibrosis of venous anastomosis

**Figure 4:**
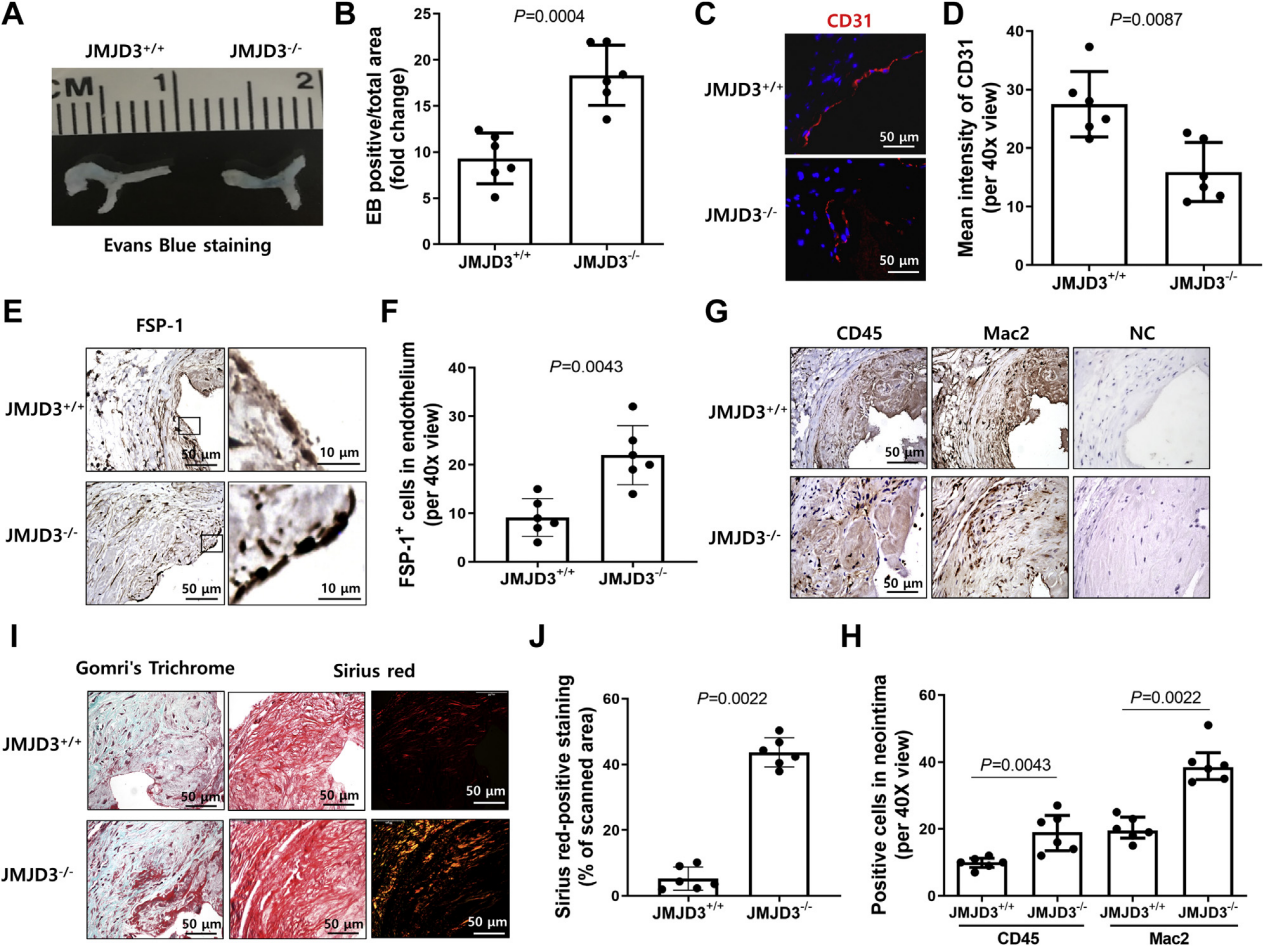
**JMJD3 deficiency in ECs is associated with increased barrier dysfunction, EndMT, inflammation, and fibrosis at venous anastomosis.***A* and *B*, at 4 weeks after placing AVF, Evans blue was administered intravenously followed by perfusion with PBS. The intensity of Evans blue leak was analyzed by ImageJ. *C* and *D*, immunofluorescent staining and intensity analysis of CD31 (*red*) in venous anastomosis of AVF from EC^JMJD3+/+^ CKD mice and EC^JMJD3 KO^ CKD mice. *E* and *F*, IHC staining of FSP-1 in venous anastomosis from EC^JMJD3+/+^ and EC^JMJD3 KO^ mice. Sections incubated with anti-rabbit secondary antibody but without primary antibody were used as negative control. Total numbers of FSP-1^+^ cell number in endothelium of venous anastomosis in AVFs were calculated. *G* and *H*, IHC staining of CD45 and Mac2 in venous anastomosis from EC^JMJD3+/+^ mice and EC^JMJD3 KO^ mice. Sections incubated with antirat or anti-rabbit secondary antibody but without primary antibody were used as negative control (NC). Total numbers of CD45^+^ or Mac2^+^ cells in neointima area in AVFs were calculated. *I*, distribution of collagen was detected by Gomori’s Trichrome staining (*red color* indicates muscle fibers, and *green/blue* color indicates collagen) and Sirius red staining (*green color* indicates type III collagen, and *red–yellow* indicates type I collagen). *J*, the ratio of area with *red–yellow* color to the whole area in Sirius red staining was calculated. There are six samples of each group. All statistical analyses between groups were performed by Mann–Whitney test. Data were represented as means ± SD. AVF, arteriovenous fistula; EC, endothelial cell; EndMT, endothelial–mesenchymal transition; FSP-1, fibroblast-specific protein 1; IHC, immunohistochemistry; JMJD3, Jumonji domain–containing protein-3.

To evaluate whether JMJD3 KO affects ECs' postsurgical reendothelialization, we examined Evans blue staining of which positive staining represents disruption of the endothelium. We found that the intensity and area of Evans blue in AVFs in EC^JMJD3 KO^ CKD mice were increased approximately threefold as compared with the levels present in AVFs in EC^JMJD3+/+^ CKD mice (Fig. 4, *A* and *B*). In addition, compared with EC^JMJD3+/+^ mice, there was a dramatic decreased signal of CD31 (Fig. 4, *C* and *D*) and a large increased signal of mesenchymal marker, fibroblast-specific protein 1 (FSP-1), in endothelium of AVFs from EC^JMJD3 KO^ mice (Fig. 4, *E* and *F*). These results indicate that endothelial JMJD3 KO delays endothelial regeneration and promotes EndMT in AVF.

Furthermore, JMJD3 KO in ECs also stimulated more robust inflammatory responses evidenced by the infiltration of macrophages (Mac-2) and monocytes (CD45) in AVF *versus* that in EC^JMJD3+/+^ mice (Fig. 4, *G* and *H*). Trichrome and Sirius red staining showed that JMJD3 KO also increased the deposition of collagens in the media and neointima (Fig. 4, *I* and *J*).

### Decreased expression of JMJD3 attenuates EC function

**Figure 5:**
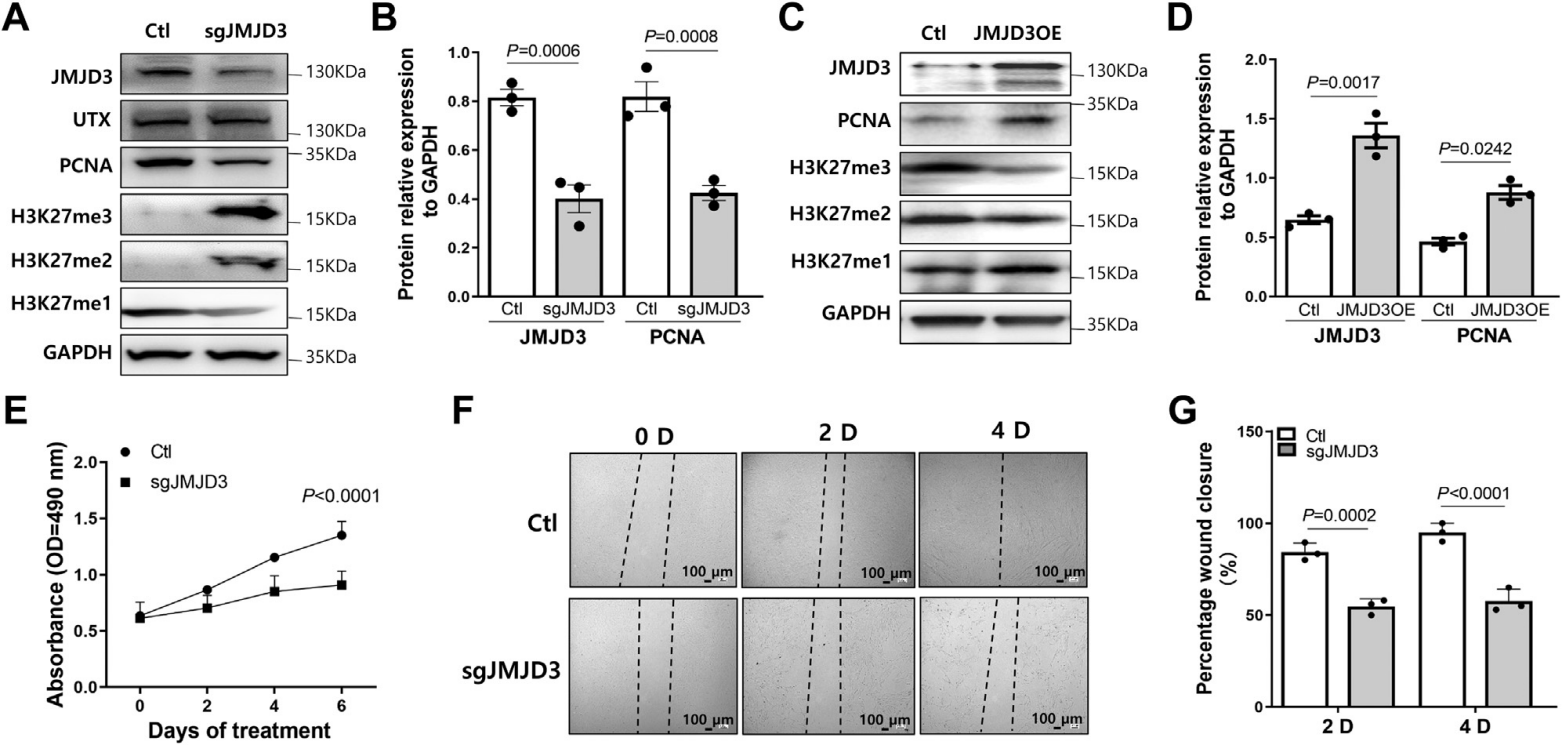
**Effects of downregulated JMJD3 on EC survival and migration.***A*, ECs infected with lentivirus-mediated sgRNA targeting JMJD3 (sgJMJD3) or nonspecific sgRNA (Ctl) for 2 days at 90% confluence in a 12-well plate. Western blot detected the expressions of JMJD3, UTX, PCNA and methylation of H3K27. Pictures are representative of three independent experiments. *B*, quantification of Western blot analyses of the expressions of JMJD3 and PCNA. Statistical significance was analyzed by one-way ANOVA. *C*, ECs infected with lentivirus-mediated overexpression of JMJD3 (JMJD3OE) for 2 days at 90% confluence in a 12-well plate. ECs infected with lentivirus packaging with empty vector were used as negative control (Ctl). Western blot was used to detect the expressions of PCNA and methylation of H3K27. Photos are representative of three independent experiments. *D*, quantification of Western blot analyses in *B*. Statistical significance was analyzed by one-way ANOVA. *E*, ECs were cultured in 96-well plate and infected with lentivirus-mediated sgRNA targeting JMJD3 (sgJMJD3) or nonspecific sgRNA (Ctl). EC proliferation was determined by MTS during different time points. Data are presented as mean ± SD from three replicates. Statistical significance between groups was analyzed by two-way ANOVA. *F* and *G*, ECs were cultured in 12-well plate and infected with lentivirus-mediated sgRNA targeting JMJD3 (sgJMJD3) or nonspecific sgRNA (Ctl). Wound-healing assay was used to detect the EC migration (*F*). The rate of migration was measured by quantifying the total distance that the cells moved from the edge of the scratch toward the center of the scratch at 2 days or 4 days when compared with 0 days (*G*). EC, endothelial cell; JMJD3, Jumonji domain–containing protein-3; MTS, 3-(4,5-dimethylthiazol-2-yl)-5-(3-carboxymethoxyphenyl)-2-(4-sulfophenyl)-2H-tetrazolium; PCNA, proliferating cell nuclear antigen; sgRNA, single-guide RNA; UTX, ubiquitously transcribed tetratricopeptide repeat, X chromosome.

To further determine the function of JMJD3 in ECs, we analyzed its effects on the proliferation and migration of ECs. Compared with control, the expression of JMJD3 in ECs was successfully knocked down with single-guide RNA (sgRNA), whereas the methylation level of H3K27 was increased (Fig. 5*A*). Expression level of ubiquitously transcribed tetratricopeptide repeat, X chromosome (UTX) was not changed by knockdown of JMJD3 (Fig. 5*A*). Furthermore, expression of the PCNA was decreased by knockdown of JMJD3 (Fig. 5, *A* and *B*), whereas PCNA level was increased by overexpression of JMJD3 (Fig. 5, *C* and *D*). The 3-(4,5-dimethylthiazol-2-yl)-5-(3-carboxymethoxyphenyl)-2-(4-sulfophenyl)-2H-tetrazolium (MTS) results showed that knockdown of JMJD3 dramatically inhibited EC proliferation (Fig. 5*E*). To examine the effect of JMJD3 knockdown on endothelial migration, wound-healing assay was performed to examine the EC migration rate. Knockdown of JMJD3 resulted in a significant decrease in wound closure when compared with control (Fig. 5*F*). Moreover, inhibition of JMJD3 activity by GSK-J4 also significantly decreased EC proliferation and migration as well as the PCNA expression (Fig. S2).

**Figure 6:**
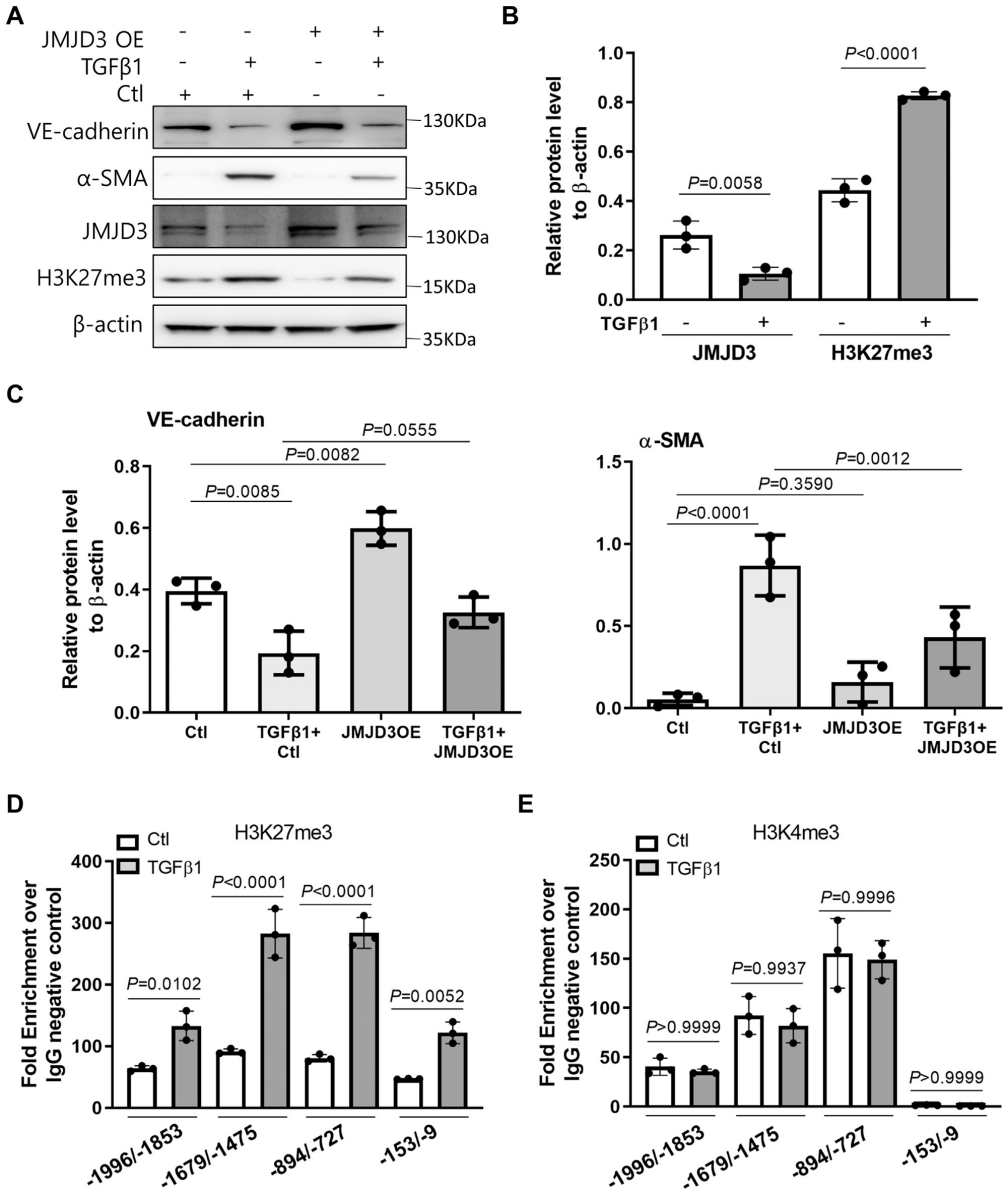
**JMJD3 mediates TGFβ1-induced EndMT.***A*, Western blot analysis of ECs overexpressing JMJD3 by lentivirus (JMJD3OE) for 2 days following treatment with 2 ng/ml TGFβ1 or solvent for another 5 days. ECs infected with lentivirus packaging with empty vector were used as negative control (Ctl). Photos are representative of three independent experiments. *B*, quantification of the expression of JMJD3 and H3K27me3 is shown as means ± SD. Statistical significance between two groups was analyzed by one-way ANOVA. *C*, quantification of the expression of VE-cadherin and α-SMA is shown as means ± SD. Statistical significance between groups was analyzed by two-way ANOVA. *D* and *E*, ECs were treated with 2 ng/ml TGFβ1 or solvent (Ctl) for 24 h. ChIP assays with rabbit anti-H3K27me3 or anti-H3K4me3 antibody were performed. Rabbit IgG was used as control. Real-time PCR amplified VE-cadherin promoter with specific primers, respectively. Quantitative PCR data were normalized to IgG negative control and displayed as fold enrichment and expressed as means ± SD. Statistical significance between groups was analyzed by one-way ANOVA. α-SMA, α-smooth muscle actin; ChIP, chromatin immunoprecipitation; EC, endothelial cell; EndMT, endothelial–mesenchymal transition; H3K27, histone H3 lysine 27; IgG, immunoglobulin G; JMJD3, Jumonji domain–containing protein-3; TGFβ1, transforming growth factor beta 1; VE-cadherin, vascular endothelial-cadherin.

Since the level of transforming growth factor beta 1 (TGFβ1) is increased in the plasma/serum of patients with kidney failure and CKD mice (34, 35, 36), and it also mediates EndMT (18), we further examined whether JMJD3 was involved in the process of TGFβ1-induced EndMT. Notably, TGFβ1 reduced the expression level of JMJD3 and induced H3K27 methylation (Fig. 6, *A* and *B*). TGFβ1 decreased the expression level of EC marker VE-cadherin and increased the expression level of mesenchymal marker α-SMA, while overexpression of JMJD3 reversed TGFβ1-mediated expression of VE-cadherin and α-SMA (Fig. 6, *A* and *C*).

Since JMJD3 overexpression upregulated the expression of VE-cadherin in ECs (Fig. 6*C*), we next determined if JMJD3 regulates the promoter activity of VE-cadherin. Chromatin immunoprecipitation–PCR (ChIP–PCR) revealed a significant increase in H3K27me3 binding to the promoter of VE-cadherin after TGFβ1 treatment (Fig. 6*D*). Because H3K27me3 was found to be enriched in the promoter region of VE-cadherin and a peak of enrichment of H3K4me3 and H3K27me3 around the transcriptional starting sites is commonly associated with bivalent genes that are marked with both H3K4me3 and H3K27me3 epigenetic modification in the same area (37), H3K4me3 enrichment in the promoter region of VE-cadherin gene was also investigated. We found that the H3K4me3 signal at the promoter of VE-cadherin was not statistically different between EC treatment with or without TGFβ1 (Fig. 6*E*). Taken together, these results suggested that JMJD3 regulated TGFβ1-induced EndMT partly through the effect of transcriptional inhibition of H3K27 methylation on the expression of endothelial markers.

### Knockdown of JMJD3 in ECs induces VSMC proliferation

**Figure 7:**
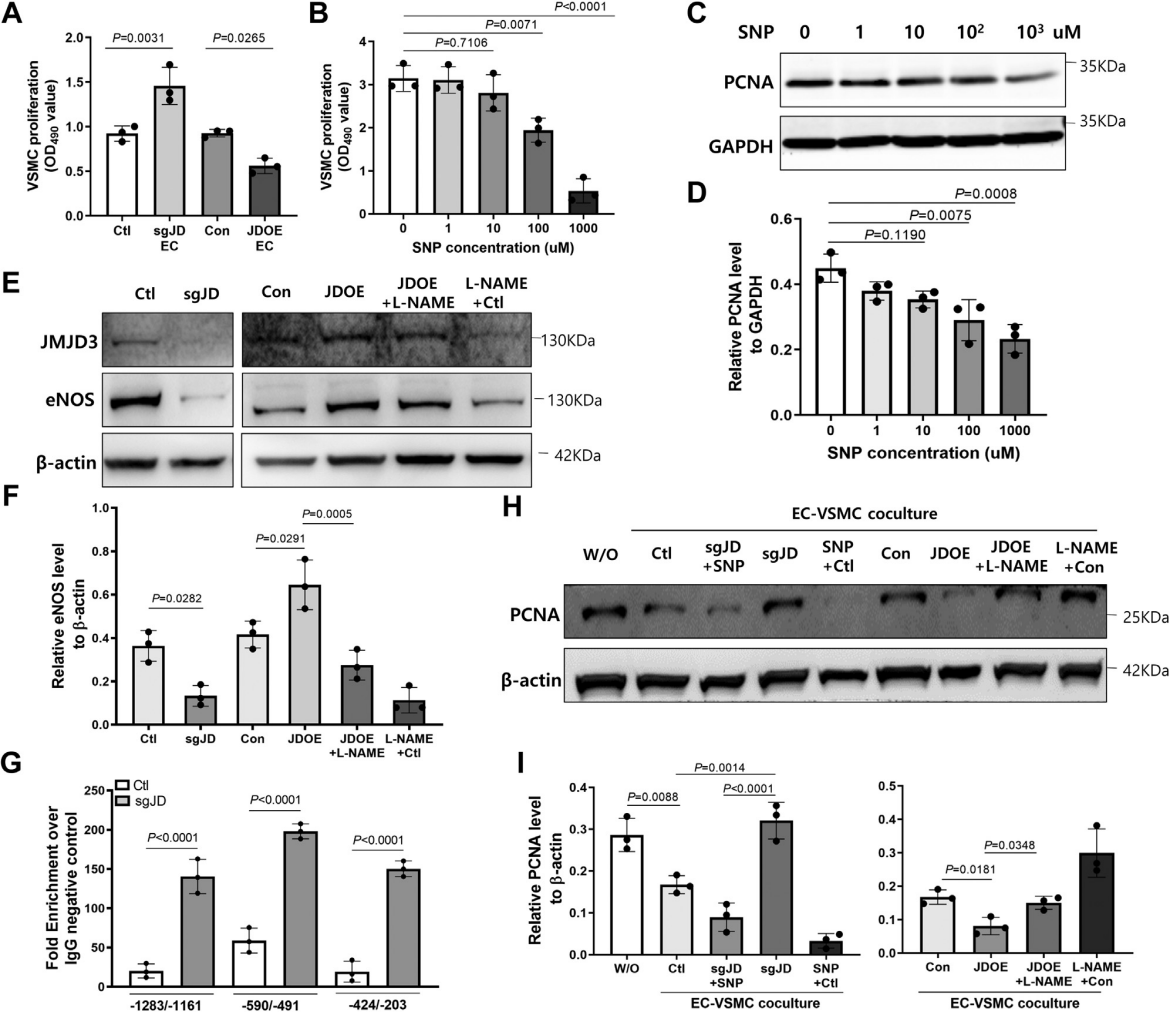
**Effects of JMJD3 knockdown in ECs on proliferation of VSMC.***A*, VSMC cells were plated on the bottom chamber of 96-well transwell cell culture system. ECs were cultured onto the top chamber of the transwell inserts and transduced with lentivirus to knockdown JMJD3 by sgRNA (sgJD) or overexpress JMJD3 (JDOE). ECs transduced with control lentivirus sgRNA (Ctl) or lentivirus packaging with empty vector (Con) were used as control. MTS assay was performed to detect cell proliferation after coculture for 72 h. Data are presented as mean ± SD for three replicates. *B*–*D*, VSMCs were cultured in 96-well plate or 12-well plate. SNP (ranged from 1 to 1000 μM) was added to treat VSMC for 48 h. VSMC proliferation was detected by MTS assay. Data are presented as mean ± SD from three replicates (*B*). PCNA expression was evaluated by Western blot (*C*) and density was quantified (*D*). *E*, ECs were cultured in 12-well plate and treated with lentivirus-mediated sgRNA targeting JMJD3 (sgJD) or overexpressed JMJD3 (JDOE) by lentivirus combined with or without L-NAME (300 μM). ECs transduced with control lentivirus sgRNA (Ctl) or lentivirus packaging with empty vector (Con) were used as control. Expression levels of JMJD3 and eNOS were detected by Western blot. Photos are representative of three independent experiments. *F*, quantification of Western blot analysis for eNOS is shown as mean ± SD. *G*, quantitative ChIP analysis of H3K27me3 at the eNOS promoter. ECs were transduced with lentivirus sgRNA targeting JMJD3 (sgJD) or control lentivirus nonspecific sgRNA (Ctl). ChIP assay was performed with rabbit anti-H3K27me3 antibodies. Rabbit IgG was used as control. Real-time PCR amplified eNOS promoter with specific primers. Quantitative PCR data were normalized to IgG negative control and displayed as fold enrichment and expressed as means ± SD. Statistical significance between groups was analyzed by two-way ANOVA. *H*, VSMCs were cocultured with ECs. VSMCs were plated on the bottom chamber of 12-well transwell cell culture system. ECs were cultured on the top chamber of transwell inserts and transduced with lentivirus to knockdown JMJD3 by sgRNA (sgJD) or overexpress JMJD3 (JDOE) with or without addition of SNP (100 μM) or L-NAME (300 μM). ECs transduced with control lentivirus sgRNA (Ctl) or lentivirus packaging with empty vector (Con) were used as control. After coculture for 48 h, VSMC lysates were used for detecting the expression levels of PCNA by Western blot. VSMC without EC coculture was also used as control (W/O). Photos are representative of three independent experiments. *I*, quantification of Western blot analysis for PCNA is shown as mean ± SD. All statistical significances between groups were analyzed by one-way ANOVA. ChIP, chromatin immunoprecipitation; EC, endothelial cell; eNOS, endothelial nitric oxide synthase; H3K27, histone H3 lysine 27; IgG, immunoglobulin G; JMJD3, Jumonji domain–containing protein-3; L-NAME, *N*(omega)-nitro-l-arginine methyl ester; MTS, 3-(4,5-dimethylthiazol-2-yl)-5-(3-carboxymethoxyphenyl)-2-(4-sulfophenyl)-2H-tetrazolium; PCNA, proliferating cell nuclear antigen; sgRNA, single-guide RNA; SNP, sodium nitroprusside; VSMC, vascular smooth muscle cell.

To investigate whether endothelial JMJD3 could regulate VSMC proliferation, primary mouse VSMCs were cocultured with ECs. MTS results revealed that knockdown of endothelial JMJD3 significantly promoted VSMC proliferation, whereas overexpression of endothelial JMJD3 inhibited VSMC proliferation (Fig. 7*A*). Since nitric oxide (NO) inhibits VSMC growth (38), we speculate that JMJD3-mediated endothelial nitric oxide synthase (eNOS)–NO in ECs regulates VSMC growth. Treatment with NO donor, sodium nitroprusside (SNP), dose-dependently inhibited VSMC proliferation and the expression of PCNA (Fig. 7, *B*–*D*). Knockdown of JMJD3 decreased eNOS expression, whereas overexpression of JMJD3 increased eNOS expression, which could be downregulated by eNOS inhibitor *N*(omega)-nitro-l-arginine methyl ester (L-NAME) (Fig. 7, *E* and *F*).

ChIP–PCR results showed a significant increase in H3K27me3 enrichment in the promoter of eNOS in ECs after knockdown of JMJD3 (Fig. 7*G*). Furthermore, overexpression of JMJD3 in ECs reduced the expression of PCNA in VSMC, and these responses could be reversed by L-NAME treatment (Fig. 7, *H* and *I*). In contrast, endothelial JMJD3 knockdown induced the expression of PCNA in VSMC, which could be blocked by SNP. Together, these findings indicated that the expression level of endothelial JMJD3 could regulate VSMC proliferation though eNOS-derived NO pathway.

### In ECs, TGFβ1 downregulates JMJD3 expression through Hes1

**Figure 8:**
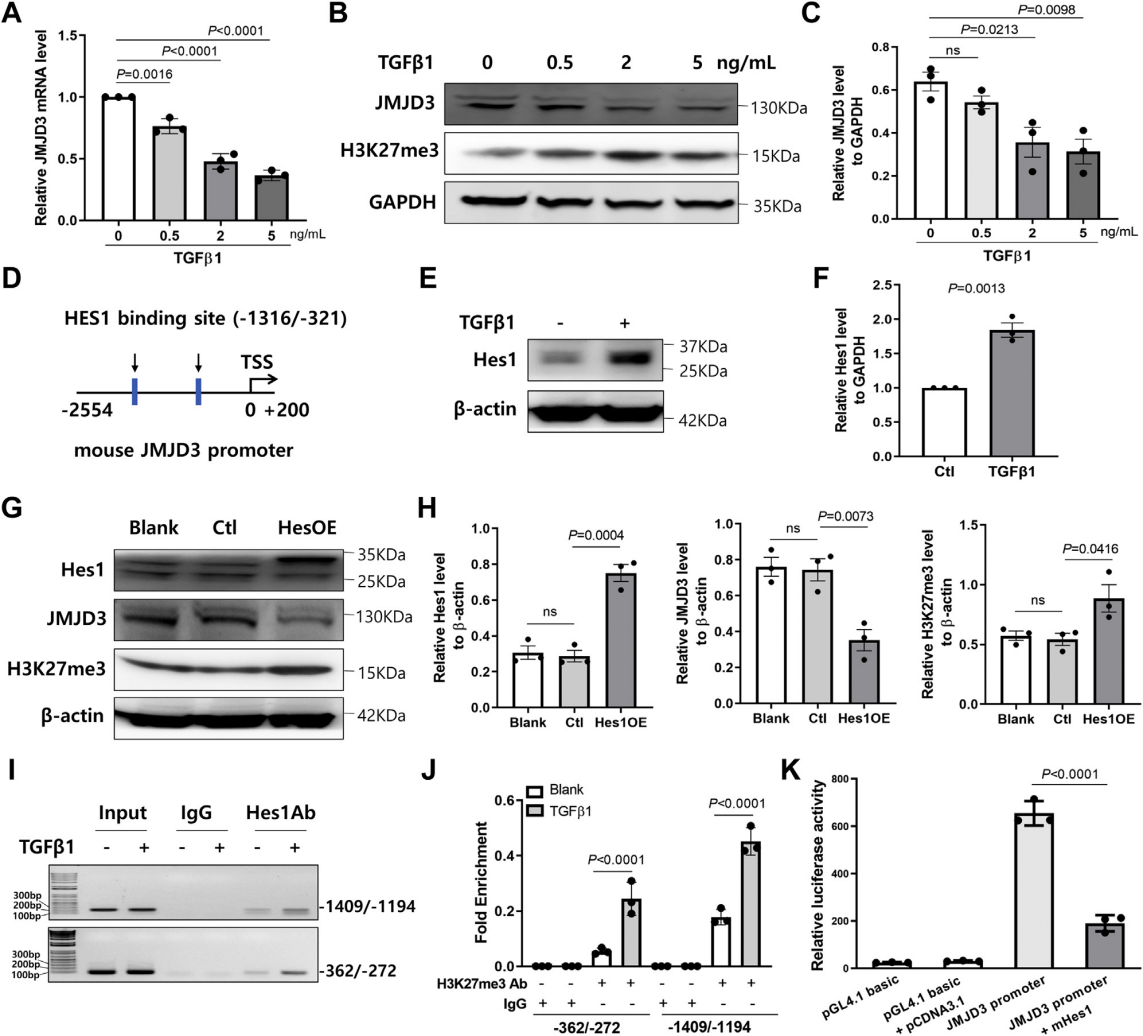
**JMJD3 is regulated by TGFβ1–Hes1 pathway in ECs.***A*, real-time PCR analysis of the mRNA level of JMJD3 after treatment of TGFβ1 or solvent (Ctl) for 24 h. Values of *JMJD3* expression level were normalized to *GAPDH*. Data are presented as mean ± SD of three independent experiments. Statistical significance was measured by one-way ANOVA. *B*, ECs were stimulated with various concentration of TGFβ1 for 48 h. The expression of JMJD3 and methylation of H3K27 was determined by Western blot. *C*, quantification of Western blot analyses for JMJD3 is shown as mean ± SD. Statistical significance between groups was analyzed by one-way ANOVA. *D*, scheme of the mouse JMJD3 locus indicating two HES1-binding sites (*vertical arrows*), which were analyzed using JASPAR. Promoter region was used in the luciferase assay. *E*, Western blot of Hes1 in EC treatment with 2 ng/ml TGFβ1 or solvent for 24 h. Photos are representative of three independent experiments. *F*, quantification of Western blot analyses for Hes1 is shown as mean ± SD. Statistical significance between groups was analyzed by Mann–Whitney test. *G*, ECs were transduced with lentivirus overexpressing Hes1 (Hes OE) or empty vector (Ctl). ECs without transduction were also used as control (blank). Western blot was performed after 2 days of culture. Photos are representative of three independent experiments. *H*, quantification of Western blot analyses for Hes1, JMJD3, and H3K27me3 is shown as mean ± SD. Statistical significance between groups was analyzed by one-way ANOVA. *I*, ChIP assays with anti-Hes1 or anti-IgG antibody from ECs with 2 ng/ml TGFβ1 or solvent (Ctl) treatment for 24 h. JMJD3 promoter was amplified with specific primers by PCR with 2% input as template for 20 cycles or with immunoprecipitated DNA as template for 35 cycles. Photos are representative of three independent experiments. *J*, quantification of PCR products for JMJD3 promoter was analyzed by ImageJ and shown as mean ± SD. Statistical significance between groups was analyzed by two-way ANOVA. *K*, HEK293T cells were cotransfected with JMJD3 promoter and Hes1 recombinant plasmid or control plasmid. Luciferase activity of the JMJD3 promoter was compared in the presence and absence of Hes1. Data are presented as mean ± SD. Spots are presented as values of three replicates. Statistical significance between groups was analyzed by two-way ANOVA. ChIP, chromatin immunoprecipitation; EC, endothelial cell; HEK293T, human embryonic kidney 293T cell line; H3K27, histone H3 lysine 27; IgG, immunoglobulin G; JMJD3, Jumonji domain–containing protein-3; TGFβ1, transforming growth factor beta 1.

The mechanisms that negatively regulated endothelial JMJD3 expression in CKD was further explored. The mRNA and protein levels of JMJD3 in ECs were suppressed dose-dependently by TGFβ1 (Fig. 8, *A*–*C*). To understand the mechanisms of TGFβ1-caused transcriptional suppression of JMJD3, the promoter region of *JMJD3* was analyzed. First, we investigated whether there were CpG islands (39) in the mouse and human *JMJD3* promoter region by New cpg report software (https://www.ebi.ac.uk/Tools/seqstats/emboss_newcpgreport/). We found that there was no potential CpG island in *JMJD3* promoter. Second, we screened the transcriptional factors in the *JMJD3* promoter. We found that there were about 109 potential transcriptional factors including hes family bHLH transcription factor 1 (Hes1) in the promoter region of mouse *JMJD3* gene (JASPAR analysis, http://jaspar.genereg.net/). Since Hes1 is a transcriptional repressor, the role of Hes1 in TGFβ1-mediated downregulation of JMJD3 was further investigated. There were two potential Hes1 consensus-binding elements found in the promoter region of mouse *JMJD3* gene (Fig. 8*D*). Western blot analyses revealed that the expression of Hes1 in ECs was increased by TGFβ1 treatment (Fig. 8, *E* and *F*). As expected, Hes1 overexpression in ECs led to a significant decrease of endogenous JMJD3 expression (Fig. 8, *G* and *H*). Furthermore, ChIP–PCR analysis revealed that there were more Hes1 bound to the *JMJD3* promoter in ECs in response to TGFβ1 (Fig. 8, *I* and *J*). As shown by luciferase reporter assays, the luciferase activity of *JMJD3* promoter was significantly inhibited by Hes1 overexpression (Fig. 8*K*).

## Discussion

Neointima formation in AVF is a complicated pathophysiological process that many types of cells and multiple signaling pathways are involved in regulating cell differentiation and activation. We and others have shown that VSMCs, bone marrow–derived FSP-1 cells, monocyte chemoattractant protein-1 positive cells, and vascular adventitial mesenchymal stem cells involve in the growth of neointima of AVFs (40, 41, 42). Multiple factors, such as oxidative stress, Notch, platelet-derived growth factor, vascular endothelial growth factor, hypoxia-inducible factor, and others, promote the migration, proliferation, and activation of VSMCs in the venous anastomosis of AVFs (43, 44, 45). ECs form a monolayer covering the inner surface of the vascular tree. This unique localization allows them to integrate physical and neurohumoral signals from the blood and surrounding tissues to regulate vascular tone, cellular adhesion, inflammation, smooth muscle phenotype, and thromboresistance. Loss of endothelium triggers the development of NIH of AVF in patients with kidney failure (46). In this study, we uncovered a signaling mechanism by which a reduction of endothelial JMJD3 expression in CKD promotes endothelial dysfunction and neointima formation in AVFs. Importantly, negative association between levels of endothelial JMJD3 and the size of neointima formation in AVFs indicated that measuring JMJD3 level in the ECs could predict the development of neointima and AVF malfunction in patients with kidney failure.

Under the CKD condition, we found that the expression level of JMJD3 in vessels, especially in ECs, was downregulated. Decreased endothelial JMJD3 expression was related to the phenotypic switching of ECs and VSMC proliferation through an eNOS–NO pathway, which was involved in vascular remodeling and neointima formation (20, 21, 47). Specific KO of endothelial *JMJD3* in mouse model resulted in more severe EndMT, vascular injury, VSMC proliferation, local extracellular matrix production, and infiltration of inflammatory cells. All these events accelerated neointima formation and AVF failure. However, our results presented here differ from the previous report showing that silence of *JMJD3* by siRNA in VSMC inhibited VSMC activation and proliferation and attenuated neointima formation in carotid injury model (13). The contradictory observations could be due to the different models (AVF in mice with kidney failure *versus* balloon injury/partial left carotid artery ligation injury in rat/mice with normal kidney function) used in the two studies. The artery injury model is induced by completely removing the endothelial layer and causing a distending mural injury in rat/mice with normal kidney function (48). In patients with kidney failure, uremic milieu contributes to a multitude of vascular diseases including venous intimal hyperplasia even prior to hemodialysis access surgery (7, 49, 50). Our studies showed that expression level of endothelial JMJD3 in vessel was significantly decreased in the context of chronic renal failure. Importantly, the results obtained from AVF in CKD mouse mimic what happens in AVFs of patients with kidney failure. Thus, we concluded that downregulated endothelial JMJD3 could induce EC dysfunction followed by VSMC proliferation and accelerate NIH in AVF.

TGFβ1, which is one of the complications of CKD, significantly inhibited JMJD3 expression in ECs. The mechanism for TGFβ1-mediated JMJD3 downregulation has never been reported before. Our findings indicated that the expression of JMJD3 in ECs can be regulated by TGFβ1–Hes1 signaling pathway. Hes1 is a transcription suppressor, which could downregulate p27^Kip1^, vascular endothelial growth factor receptor 2/3 and DLL4 (delta-like canonical Notch ligand 4), and control cell proliferation, survival, and differentiation (51, 52). We found that more Hes1 bound to the *JMJD3* promoter upon treatment of ECs with TGFβ1. Overexpression of Hes1 inhibited the transcriptional activity of *JMJD3* promoter. Therefore, endothelial JMJD3 was downregulated by TGFβ1–Hes1 pathway in CKD *via* binding of Hes1 to its gene promoter.

**Figure 9:**
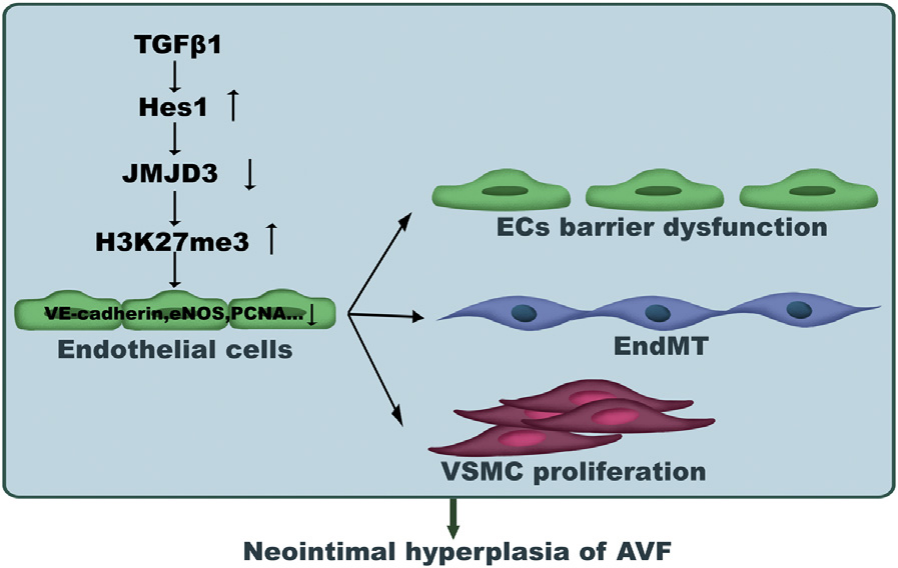
**Schematic showing that decreased endothelial JMJD3 regulates neointima hyperplasia of AVFs.** In CKD, Hes1 expression is induced by TGFβ1 signaling in ECs. Hes1 binds to the *JMJD3* promoter and inhibits *JMJD3* transcription. Decreased JMJD3 expression epigenetically reduces transcription of genes (such as VE-cadherin, eNOS and PCNA, and others) leading to EC barrier dysfunction and EndMT. Moreover, decreased JMJD3 in ECs also suppresses eNOS-NO production, promoting VSMC proliferation. These responses result in inflammation and neointima hyperplasia in AVF. AVF, arteriovenous fistula; CKD, chronic kidney disease; EC, endothelial cell; EndMT, endothelial–mesenchymal transition; eNOS, endothelial nitric oxide synthase; JMJD3, Jumonji domain–containing protein-3; NO, nitric oxide; PCNA, proliferating cell nuclear antigen; TGFβ1, transforming growth factor beta 1; VE-cadherin, vascular endothelial-cadherin; VSMC, vascular smooth muscle cell.

In conclusion, we have identified a novel role of endothelial JMJD3 in developing neointimal formation in creating AVFs through regulation of EC function and VSMC proliferation (Fig. 9). Endothelial JMJD3 expression was downregulated through TGFβ1–Hes1–JMJD3 axis in CKD. Reduced expression of JMJD3 suppressed the transcription of EC markers and delayed EC regeneration leading to EndMT and endothelial barrier dysfunction. Decreased JMJD3 in ECs also inhibited eNOS expression and NO production, which promoted VSMC proliferation. These responses resulted in neointimal formation in AVF. Moreover, decreased expression of JMJD3 in ECs was associated with the increased NIH in created AVF of patients with kidney failure. Thus, targeting endothelial JMJD3 by increasing its expression may have clinical implications for therapeutic selection to attenuate NIH and stenosis in AVF of kidney failure patients.

## Experimental procedures

### Generation of EC-specific JMJD3 KO mice

All experiments involving animals were approved by the Institutional Animal Care and Use Committee of Baylor College of Medicine and were performed in accordance with the National Institutes of Health guidelines. Mice were housed in a conventional animal facility on a 12 h light/12 h dark regimen and fed a regular chow ad libitum. JMJD3^flox/flox^ (JMJD3^f/f^) mice were kindly provided by Dr Wang (Houston Methodist Research Institute). JMJD3^f/f^ mice were backcrossed with C57BL/6 mice for at least 10 generations and identified by genotyping using specific primers (Table 1) as described previously (53). VE-cadherin-CreER^T2^ (VE-CreER^T2^) transgenic mice (C57BL/6) were obtained from Dr Thuy (Baylor College of Medicine) after permission from Dr Adams (Cancer Research UK) and identified by genotyping with specific primers (Table 1). Mice carrying floxed JMJD3 allele were bred with VE-CreER mice to generate JMJD3^f/f^/VECreER^+^ mice (EC^JMJD3 KO^). EC-specific JMJD3 deletion was induced by tamoxifen (dissolved in corn oil, i.p., 40 mg/kg body weight, for 5 consecutive days) at fourth week after the mice were born. JMJD3^f/f^/VECreER^−^ mice (EC^JMJD3+/+^) treated with tamoxifen were used as control.

**Table 1:** List of primer sequences used for genotyping, plasmid construction, or ChIP–PCR

Usage	Genes	Primer sequences
Genotyping	JMJD3 flox	F: 5′-TGGACATTCTCTCCTCCCAG-3′ R1: 5′-GCGTATTTAGCGATGGTGGT-3′ R2: 5′-AGGGTGGGTGTTGAGACAAG-3′
	VE-cadherin Cre	F: 5′-GTTCGCAAGAACCTGATGGACA-3′ R: 5′-CTAGAGCCTGTTTTGCAGGTTC-3′
Plasmid construction	JMJD3 (Promoter)	F (KpnI): 5′-ATCG*GGGTACCC*TTGCCCCATTCTCCTCGTAGCAG-3′ R (EcoRV): 5′-CGG*ATCGAT*ATCAGACTTCTCTATCCACAGAAAGC-3′
	JMJD3 (sgRNA)	F: 5′-CACCGACAAAAGTACTGTTACCGG-3′ R: 5′-AAACCCGGTAACAGTACTTTTGTC-3′
	Hes1 (ORF)	F (BamHI): 5′-CGC*GGATCC*ATGCCAGCTGATATAATGGAG-3′ R (EcoRI): 5′-CCG*GAATTC*TCAGTTCCGCCACGGTCTCCAC-3′
	Hes1 (sgRNA)	F: 5′-CACCGGACCGGGCCGCTGTGAGCGA-3′ R: 5′-AAACTCGCTCACAGCGGCCCGGTCC-3′
Real-time PCR	JMJD3	F: 5′-CCCCCATTTCAGCTGACTAA-3′ R: 5′-CTGGACCAAGGGGTGTGTT-3′
	GAPDH	F: 5′-AGTGGGAGTTGCTGTTGAAATC-3′ R: 5′-TGCTGAGTATGTCGTGGAGTCTA-3′
ChIP-PCR	eNOS (promoter)	F1: 5′-GCCAGGCACCTGAAAGAA-3′ R1: 5′-AAGTAGGGAGCCACCTATCC-3′ F2: 5′-GGACACTTGACAGGATTGGAG-3′ R2: 5′-ATCTCAATTTCCTGAACCCACA-3′ F3: 5′-TCCCAAAGGACTCTGAGATAGG-3′ R3: 5′-TGAGGGCTAGAGCTGATAAGT-3′
	VE-cadherin (promoter)	F1: 5′-TTGGGACTGAACTTCTGCTATG-3′ R1: 5′-TACTCTAGCCCTGGTGGATATT-3′ F2: 5′-GGCAGGGAACAGAACAGATT-3′ R2: 5′-AGGACATCAGAGTAAGGAGAGAG-3′ F3: 5′-CCCGAGTGCTGGGATTAAAG-3′ R3: 5′-GGAGATAGCCATAGGTCTGAGT-3′ F4: 5′-CTCACAAAGGAACAATAACAGGAAA-3′ R4: 5′-CAGGGCCGAGCTTTGTG-3′
	JMJD3 (promoter)	F1: 5′-TAGGGTGACCTGGGTACTTTAG-3′ R1: 5′-GAGGGCCAACAGCCATTTA-3′
		F2: 5′-GTCTTGTTAGGGTTGGAGGTAG-3′ R2: 5′-GGGACATCTACTGTCAAGCAA-3′

Abbreviations: F, forward primer; R, reverse primer.

Note: Restriction enzyme sites in primers are shown in italic.

### Mouse CKD and AVF models

CKD of EC^JMJD3 KO^ mice and EC^JMJD3+/+^ mice (male, 10 weeks old; female, 12 weeks old) was performed by subtotal nephrectomy as previously described (54). Mice were anesthetized with ketamine (125 mg/kg body weight) and xylazine (6.4 mg/kg body weight) through intraperitoneal injection. For the sham surgery, the same surgery procedures were performed but without nephrectomy. BUN levels in blood samples collected from the tail vein were measured by the urease method (55).

After 6 to 7 weeks of last nephrectomy, AVF was constructed in EC^JMJD3+/+^ and EC^JMJD3 KO^ mice by connecting the venous end of the external jugular vein to the side of the common carotid artery as previously described (56, 57). Briefly, after mice were anesthetized, the dorsomedial branch of the left external jugular vein and common carotid artery were individually dissected from its perivascular tissue bluntly and completely under a dissecting microscope (Zeiss). A longitudinal incision in the middle of the artery of approximately 1 mm using the specialized microscissor was made, and the vein was cut using a scissor just proximally to the ligation. During surgery, heparin was used as anticoagulant. A unilateral venous end to arterial side anastomosis was created with an interrupted suture. After unclamping, patency was confirmed visually. The mice were kept warm, and the analgesia (buprenorphine) was provided before surgery. AVF surgeries were performed in seven EC^JMJD3+/+^ mice with CKD (four males and three females) and nine EC^JMJD3 KO^ mice with CKD (five males and four females), of which one mouse in each group died before sample collection (one female in wildtype group and one male in KO group). Samples were collected at 4 weeks after AVF creation. Operators for all animal experiments were blinded to group allocation during all analytical procedures.

### Evans blue examination of endothelial barrier function of AVFs

At fourth week after AVF surgery, the mice were anesthetized with ketamine and xylazine. Total 50 μl of 5% Evans blue (Sigma–Aldrich; catalog no.: E2129) diluted in saline was injected into the right external jugular vein and kept for 5 min. And then the mice were euthanized by perfusion of the right ventricle with 10 ml PBS and then 10 ml of 10% formalin and kept for 10 min, respectively. After the mice were euthanized, AVFs were removed and photographed. The area of Evans blue staining was semiquantified using ImageJ software (National Institutes of Health) as previous reports (58, 59).

Morphology of AVFs was examined after paraffin embedding. The area of neointima and media were defined as the regions between the lumen and the adventitia. The vessel wall thickness was measured by NIS-Elements BR 3.0 program (Nikon) as area of the vessel minus that of the lumen. Five slides of cross sections were obtained by selecting the first of every 10 sections from each AVF and were used to evaluate neointima formation in AVFs.

### AVF samples of patients with kidney failure

Anastomotic vein of the AVF samples was collected prospectively from 16 patients with kidney failure who first underwent staged brachiobasilic transposition AVF creation at Houston Methodist Hospital between July 2014 and June 2017, which was approved by the Institutional Review Board. At the first-stage procedure, the basilic vein was divided and anastomosed to the brachial artery. Four weeks later, a second procedure was performed for transposition of the vein to a more superficial location away from nerves and the brachial artery to facilitate easier needle access for dialysis. During this procedure, the vein was mobilized from the original anastomosis to the axilla, divided near the anastomosis, retunneled in a more anterior aspect of the arm, and a new anastomosis performed to the brachial artery. A 5 mm sample portion of the original anastomotic vein was collected. Specimens were placed immediately into formalin and then embedded for histologic and immunostaining analysis. Among the 16 AVF samples collected in the secondary stage of the surgery, eight AVFs showed >80% of an intact circumferential structure, and the samples with intact circumferential structure were included for further study (Table 2).

**Table 2:** Demographic and AVF characteristics of patients with kidney failure

Sample no.	Age (year)	Gender	Circumferential structure	Ratio of neointima to lumen of AVF	Time between two-stage procedure (day)	Vein diameter 2 cm above anastomosis (cm)	PSV ratio >3	Using AVF at 12 months
3	38	M	>80%	5.66	35	0.43	No	Yes
10	50	F	100%	2.19	42	0.6	No	Yes
14	65	M	100%	2.99	21	0.32	Yes	Yes
18	53	M	100%	24.09	42	0.71	No	No
24	32	F	100%	1.07	42	0.35	No	No
44	59	F	100%	2.35	21	0.45	No	Yes
56	52	M	>80%	0.25	42	0.51	Yes	Yes
60	55	F	100%	1.95	28	0.67	Yes	Yes

Abbreviations: F, female; M, male; PSV, peak systolic velocity.

Note: Vein diameter 2 cm above anastomosis (cm) is determined by intraoperative ultrasound.

### Histology, morphometry, and immunostaining

Tissue sections (4 μm) at 1 to 2 mm from the anastomosis of AVFs were used for histology, morphometry, and immunostaining analysis. Hematoxylin and eosin staining and Gomori’s trichrome (Polysciences, Inc; catalog no.: 24205) staining were performed according to the manufacturer’s instructions and viewed with bright-field light microscopy. Sirius Red staining was performed by incubating slides in 0.1% Sirius Red F3B (Sigma–Aldrich; catalog no.: 365548) for 1 h, washing twice in acidified water, dehydrating thrice in 100% ethanol, and then clearing in xylene. Sirius Red–stained sections were illuminated with a polarized light microscopy. For immunohistochemistry, sections were deparaffinized, rehydrated, and retrieved with 10 mM citrate buffer (pH 6.0). Subsequently, they were treated with 3% H_2_O_2_. After washing with PBS and blocking, sections were incubated with the primary antibodies: rabbit anti-α-SMA monoclonal antibody (Cell Signaling Technology; catalog no.: 19245), rabbit anti-fibrinogen monoclonal antibody (Abcam; catalog no.: ab92572), rat anti-CD45 monoclonal antibody (BD Biosciences; catalog no.: 553076), rat anti-Mac-2 monoclonal antibody (Cedarlane Laboratories; catalog no.: CL8942LE), rabbit anti-FSP-1 polyclonal (DAKO; catalog no.: A5114), or rabbit anti-PCNA polyclonal antibody (Santa Cruz; catalog no.: sc-7907). Staining was performed according to the ABC kit instructions (Vector Laboratories; catalog nos.: PK-6104 and PK-6101). Sections incubated with anti-rabbit or anti-rat secondary antibody but without primary antibody were used for control. Signals were visualized using a peroxidase substrate DAB kit (Vector Laboratories; catalog no.: SK-4100), and photographs were recorded using the NIS-Elements BR 3.0 program. Staining positive signals from images (×40) of each section were analyzed using H-score method through Immunohistochemistry Profiler or counted directly from Analyze Particles with Color Deconvolution plugin of ImageJ software (60, 61).

Digital images of hematoxylin and eosin–stained or immunohistochemical α-SMA-stained venous sections were used for measuring morphometric parameters. Area measurements were carried out by analyzing the lumen area and neointima area of complete circumferential structure in visual field, which were enclosed by lines and performed using NIS-Elements BR 3.0 program. Lumen area was measured by tracing around the edge of the lumen space. Neointimal area was measured by tracing around the internal elastic lamina, then subtracting the lumen area as previous reports (11, 49).

For double immunofluorescence staining, the sections were deparaffinized, rehydrated, and treated with antigen retrieval. After blocking, primary antibodies were added for overnight at 4 °C, followed by incubating with Alexa Fluor–conjugated secondary antibodies (Invitrogen) at room temperature for 30 min. 4′,6-Diamidino-2-phenylindole (SouthernBiotech; catalog no.: 0100-20) was used to stain nuclear DNA. Rabbit anti-JMJD3 (Abcam; catalog no.: ab38113), rabbit anti-H3K27me3 antibody (Abclonal; catalog no.: A2363), rabbit anti-α-SMA antibody (Sigma; catalog no.: A5228), and rabbit anti-fibrinogen antibody (Abcam; catalog no.: ab92572) were used as primary antibodies. Rat anti-CD31 (Dianova; catalog no.: DIA-310) or mouse anti-vWF (anti-von Willebrand factor; Abcam; catalog no.: ab6994) were used to stain ECs. To capture images, the Nikon Eclipse 80i fluorescence microscope and NIS-Elements BR 3.0 program was used. Images from each section were analyzed, and positive signals in a region of interest were quantified using ImageJ software following a detailed report (62). Briefly, we used the “Hyperstack” and “Colorized” options to analyze each of fluorescent channels collected in the original experiments. The drawing pen was used to circle the area of the tissue to be quantitated, and the mean intensity value was measured.

### Primary EC and VSMC isolation and culture

Mouse ECs from lung were isolated as described previously (14). ECs were cultured in high-glucose Dulbecco's Modified Eagle's Medium (DMEM) with 16% fetal bovine serum (FBS) (Gibco; catalog no.: 26140079), 15% EC growth supplement (Sigma; catalog no.: E2759), 1% sodium pyruvate (Sigma; catalog no.: S8636), 0.6% nonessential amino acids (Carlson; catalog no.: NAL03), and 0.1% fungizone (Gibco; catalog no.: 15290-018). The purified ECs were confirmed by immunostaining with rat anti-VE-cadherin antibody (Santa Cruz; catalog no.: sc-28644). Over 95% of these cells were positive for VE-cadherin.

Primary VSMCs were isolated from mouse aorta (63) and cultured in DMEM with 10% FBS. The purified VSMCs were confirmed by immunostaining with rabbit anti-α-SMA antibody (Sigma; catalog no.: A5228). Over 95% of these cells were positive for α-SMA.

### Coculture of VSMCs and ECs in transwell system

For coculture of VSMCs and ECs, VSMCs were plated on the bottom chamber of transwell cell culture system (pore size 0.4 μm; CoStar Corp) using DMEM with 10% FBS. The next day, the VSMCs were washed and incubated in serum-free media overnight. ECs were cultured on the top chamber of the transwell and allowed to grow until confluence and then placed into the 12-well or 96-well plate of which bottom contained VSMC to initiate the experiments. ECs were transduced with lentivirus to knockdown or overexpress JMJD3 or lentivirus packaging with empty vector used as control. After 48 h, ECs were cultured in fresh EC medium with 2% FBS in addition of 100 μM SNP (MedChemExpress; catalog no.: 14402-89-2) or 300 μM L-NAME (MedChemExpress; catalog no.: HY-18729A) for another 48 h.

### Cell migration and proliferation

Wound-healing assay was performed to quantify the EC migration. Primary ECs were grown in a 12-well plate as confluent monolayer and transfected with lentivirus-mediated sgRNA targeting JMJD3 or nonspecific sgRNA control for 48 h and then made quiescent for 8 h in medium containing 2% FBS. A scratch wound was created physically using a sterile pipette tip. The cells were washed with phosphate-buffered saline to remove the debris and further cultured in EC medium containing 16% FBS. The healing of the scratch wound was monitored at different time points by phase-contrast microscopy and photographed. The rate of migration was measured by quantifying the total distance that the cells moved from the edge of the scratch toward the center of the scratch. The experiments were repeated three times independently.

MTS assay was used to examine cell proliferation. In brief, primary ECs or VSMCs were seeded on 96-well plates (5000 cells/well) as confluent monolayer. For ECs, cells were transfected with lentivirus-mediated sgRNA targeting JMJD3 or nonspecific sgRNA control for 48 h and quiescent for 8 h in medium containing 2% FBS. ECs were then incubated with EC medium. For VSMCs, cells were quiescent for 8 h in medium without FBS and then cocultured with ECs in transwell system or incubated with medium containing various concentration of SNP (MedChemExpress; catalog no.: 14402-89-2). Cell proliferation during growth periods was evaluated by the MTS method with the Cell Titer 96 Aqueous nonradioactive cell proliferation assay kit (Promega; catalog no.: G5421) according to the manufacturer's instruction. The 96-well plate was incubated at 37 °C for 1 h after the addition of assay solution (20 μl). Subsequently, the absorbance was measured at 490 nm by an ELISA reader (FLUOstar Omega; BMG Labtech).

### Lentivirus construction

The ORF of mouse JMJD3 constructed into lentivirus vector was obtained from Addgene. The ORF of mouse Hes1 was constructed into pCDH-CMV-MCS-EF1-GFP-Puro vector (System Biosciences; catalog no.: CD513B-1) by BamHI and EcoRI double enzyme digestion. The guide RNA targeting mouse JMJD3 or Hes1 was constructed into CRISPR–Caspase9 vector, respectively. The packaging vectors VSVG and Δ8.9 for lentivirus were purchased from Addgene. The primers used for vector construction and guide RNA information have been shown in Table 1.

To produce lentivirus, human embryonic kidney 293T cells were transfected with a lentiviral plasmid expressing sgRNA/complementary DNA together with the packaging plasmids VSVG and Δ8.9 using calcium phosphate transfection reagent. Lentivirus packaging with nonspecific sgRNA or empty vector was used as control. Viral supernatants were collected at 24 and 48 h after transfection, respectively. Viral supernatants were further concentrated by ∼200-fold using ultracentrifugation at 25,000 rpm for 2 h at 4 °C.

### Plasmid construction

Luciferase reporter plasmids were constructed as follows: promoter sequence of JMJD3 (−2000 to +200 bp) was amplified from mice genomic DNA by PCR using primers (Table 1). The PCR fragments were cloned into pGL4.1 promoter vector (Promega; catalog no.: E6651) after cutting with KpnI or EcoRV. ORF DNA fragment of Hes1 was amplified from mouse aorta by RT–PCR using primers containing BamHI and EcoRI restriction sites (Table 1) and cloned into pCDNA3.1(+) vector (Invitrogen; catalog no.: V790-20). The isolated clones were confirmed by DNA sequencing.

### Transient transfections and luciferase activity assays

Human embryonic kidney 293T cells were seeded into 12-well plates at a density of 2 × 10^5^ cells per well overnight before transfection. The pGL4.1-JMJD3 promoter plasmid (500 ng) and pRL-TK (25 ng) (Promega) were cotransfected with either pcDNA-Hes1 (500 ng) or an empty vector (500 ng) using Lipofectamine 2000 (Invitrogen). The plasmid pRL-TK was used as an internal control to normalize differences in transfection efficiency. After 48 h of incubation, cells were lysed and luciferase activities were measured using a dual-luciferase assay system (Promega) on an Infinite F500 microplate reader (Tecan). For each plasmid construct, the experiment (three replicates per experiment) was independently repeated three times.

### ChIP–PCR

ChIP assay was performed in ECs after treatment with 2 ng/ml TGFβ1 (R&D Systems; catalog no.: 7754-BH) for 24 h or in ECs after transfection with lentivirus-mediated sgRNA targeting JMJD3 for 48 h. ChIP assay was performed using Protein A/G plus agarose beads from Santa Cruz according to the manufacturer’s protocol (https://www.scbt.com/zh/resources/protocols/chromatin-immunoprecipitation-assays). Briefly, after wash with PBS, cells were crosslinked with 1% formaldehyde solution for 10 min at room temperature and quenched with 0.125 M glycine for 5 min. Cells were rinsed twice with cold PBS containing protease inhibitor cocktail (Roche). The cells were then resuspended and lysed in nuclear lysis buffer (1× PBS, 1% NP-40, 0.5% sodium deoxycholate, and 0.1% SDS) containing protease inhibitor cocktail and sonicated to solubilize and shear crosslinked DNA to 200 to 500 bp. The resulting chromatin extract was incubated overnight at 4 °C with 2 μg anti-Hes1 (Santa Cruz; catalog no.: sc-13844) or anti-H3K27me3 (Millipore; catalog no.: 07449) or H3K4me3 (Millipore; catalog no.: 07473) antibody. Goat or rabbit isotype immunoglobulin G was used as negative control. Next day, each sample was added 20 μl Protein A/G plus agarose beads (Santa Cruz; catalog no.: sc2003) and then incubated at 4 °C for 2 h. Beads were washed with radioimmunoprecipitation assay lysis buffer. The complexes were eluted from beads in elution buffer by heating at 65 °C with occasional vertexing over 2 h, and crosslinks were reversed by overnight incubation at 65 °C. Input DNA (reserved from sonication) was concurrently treated for crosslink reversal. DNA was treated with proteinase K and purified.

The primers used in PCR can be found in Table 1. PCR with high-fidelity DNA polymerase (NEB) or SYBR Green quantitative PCR (Bio-Rad) was performed on immunoprecipitation eluates, 2% chromatin input not subjected to immunoprecipitation, which was used as reference. Relative fold enrichment was calculated by determining the immunoprecipitation efficiency (ratios of the amount of immunoprecipitated DNA to that of the input sample).

### Western blot

The protein content of cell extracts or tissue prepared in radioimmunoprecipitation assay lysis buffer was determined using the Bradford protein assay kit (Bio-Rad). About 30 μg of proteins were separated by SDS-polyacrylamide gel electrophoresis. After transferring to nitrocellulose membranes, immunoblots were probed separately with various primary antibodies. Subsequently, the immunoblots were blocked with 5% skimmed milk in Tris-buffered saline solution. Fluorescently labeled or horseradish peroxidase–conjugated secondary antibodies were detected by the Odyssey Infrared Imaging System (LI-COR Biosciences). Primary antibodies were used as following: rabbit anti-JMJD3 (Millipore; catalog no.: 07-1533), rabbit anti-H3K27me3/me2/me1 (Abclonal; catalog nos.: A2363, A2362, and A2361), rabbit anti-UTX (GeneTex; catalog no.: GTX12146), rabbit anti-eNOS (Cell Signaling Technology; catalog no.: 32027), rabbit anti-VE-cadherin (Santa Cruz; catalog no.: sc-28644), mouse anti-α-SMA (Sigma; catalog no.: A5228), goat anti-Hes1 (Santa Cruz; catalog no.: sc-13844), rabbit anti-PCNA (Santa Cruz; catalog no.: sc-7907), mouse anti-β-actin (GeneTex; catalog no.: GTX629630), and mouse anti-GAPDH (Santa Cruz; catalog no.: sc-32233).

### Quantitative RT–PCR analysis

ECs were treated with TGF-β1 for 24 h and washed with cold PBS buffer. Total RNA was extracted by Trizol reagent (Invitrogen; catalog no.: 15596026) according to the manufacturer's instructions. Complementary DNA was prepared from 1 μg of total RNA using Reverse transcription reagents (Bio-Rad; catalog no.: 1708841). Quantitative PCR was performed using Power SYBR Green PCR Master Mix (Bio-Rad; catalog no.: 1708882), and the detection was carried out in a CFX96 Real-Time PCR System (Bio-Rad). All the values of the target gene expression level were normalized to GAPDH, and the 2^−△Ct^ method (2^[-(Ct^_sample_^-Ct^_GAPDH_^)]^) was used to calculate the relative expression of target genes (64). The mean minimal cycle threshold values (Ct) were calculated from three independent reactions. The primers used in real-time PCR can be found in Table 1.

### Statistics

All statistical analyses performed at least three independent biological or experimental replicates. Statistical analyses were performed in GraphPad Prism, version 8 (GraphPad Software, Inc). Scatter dot plots and error bars represent the mean ± SD or median with interquartile range. Significant differences were determined by Mann–Whitney test and unpaired Student’s *t* test when comparing two groups, one-way or two-way ANOVA when comparing multiple groups. Association between two variables was analyzed by Pearson's correlation. Statistical tests were described in each figure legend. Differences were considered statistically significant at *p* value < 0.05.

## Data availability

All representative data are contained within the article.

## Supporting information

This article contains supporting information.

## Conflict of interest

The authors declare that they have no conflicts of interest with the contents of this article.
