# Supplementary material for: Downregulation of the endothelial histone demethylase JMJD3 is associated with neointimal hyperplasia of arteriovenous fistulas in kidney failure
Source: J Biol Chem. 2022 Mar 10;298(5):101816. doi: 10.1016/j.jbc.2022.101816 (PMC9052161; doi:10.1016/j.jbc.2022.101816)
Supplement: Supplemental Figures S1 and S2 [file mmc1.docx]

**Downregulation of the endothelial histone demethylase JMJD3 is associated with neointimal hyperplasia of arteriovenous fistulas in kidney failure**

Shaozhen Feng^1,2^, Eric K. Peden^3^, Qunying Guo^2^, Tae Hoon Lee^2^, Qingtian Li^2^, Yuhui Yuan^4^, Fengzhang Huang^2^, Jizhong Cheng^2,^*

**Supporting information:**

**1. Supporting figures**

**1. Supporting Figures**


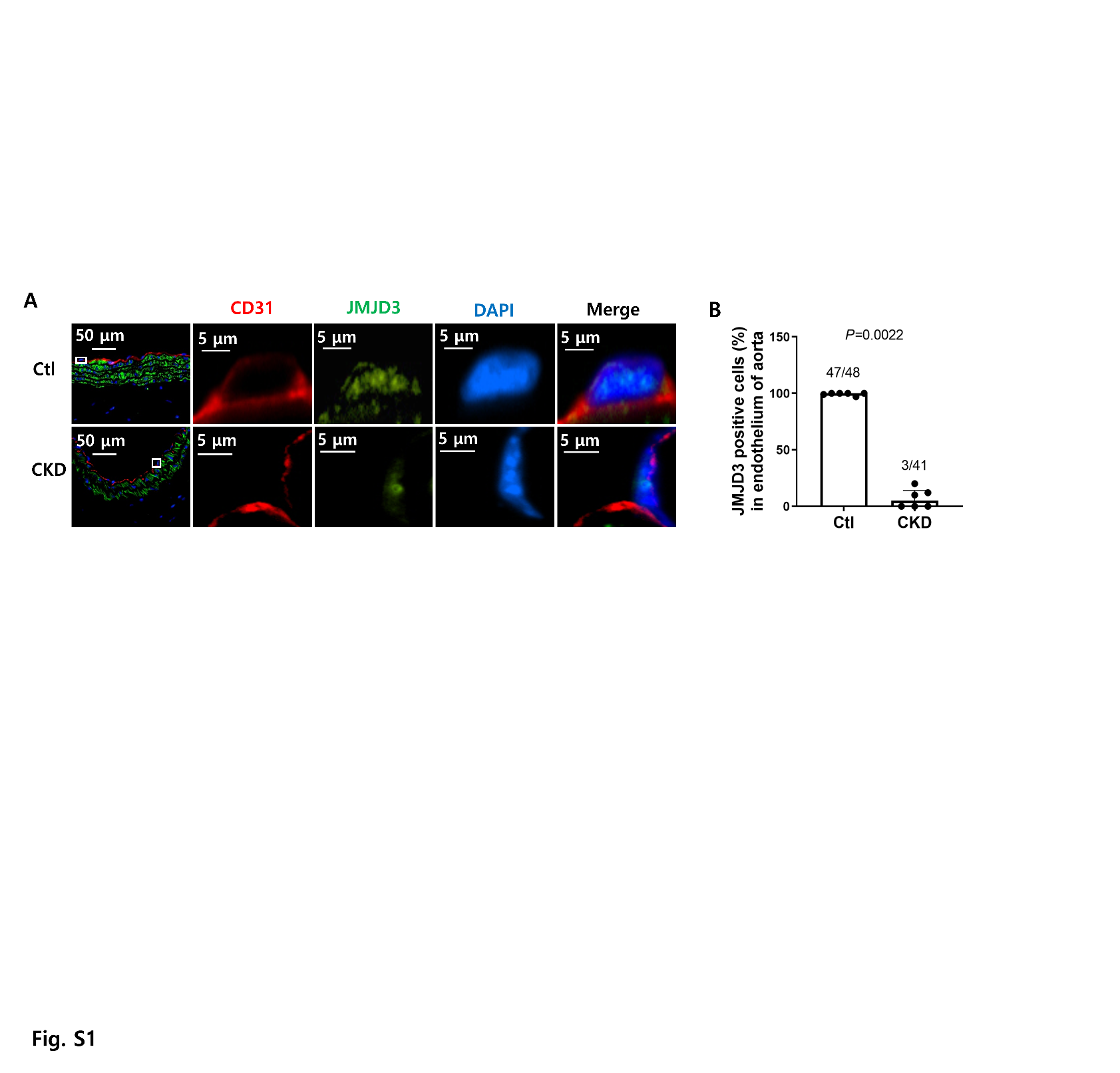


Figure S1. Expression of endothelial JMJD3 in aorta of CKD mice. A. Immunofluorescent staining of JMJD3 (green) and CD31 (red) in aortas from control (Ctl) and CKD mice. The boxed region in the left panel is enlarged in the right panels. B. Percentage of JMJD3 positive cells in total CD31 positive cells of aortas was calculated in control (n = 6) and CKD mice (n = 6). Data were represented as mean ± SD. Statistical significance was measured using Mann-Whitney test.


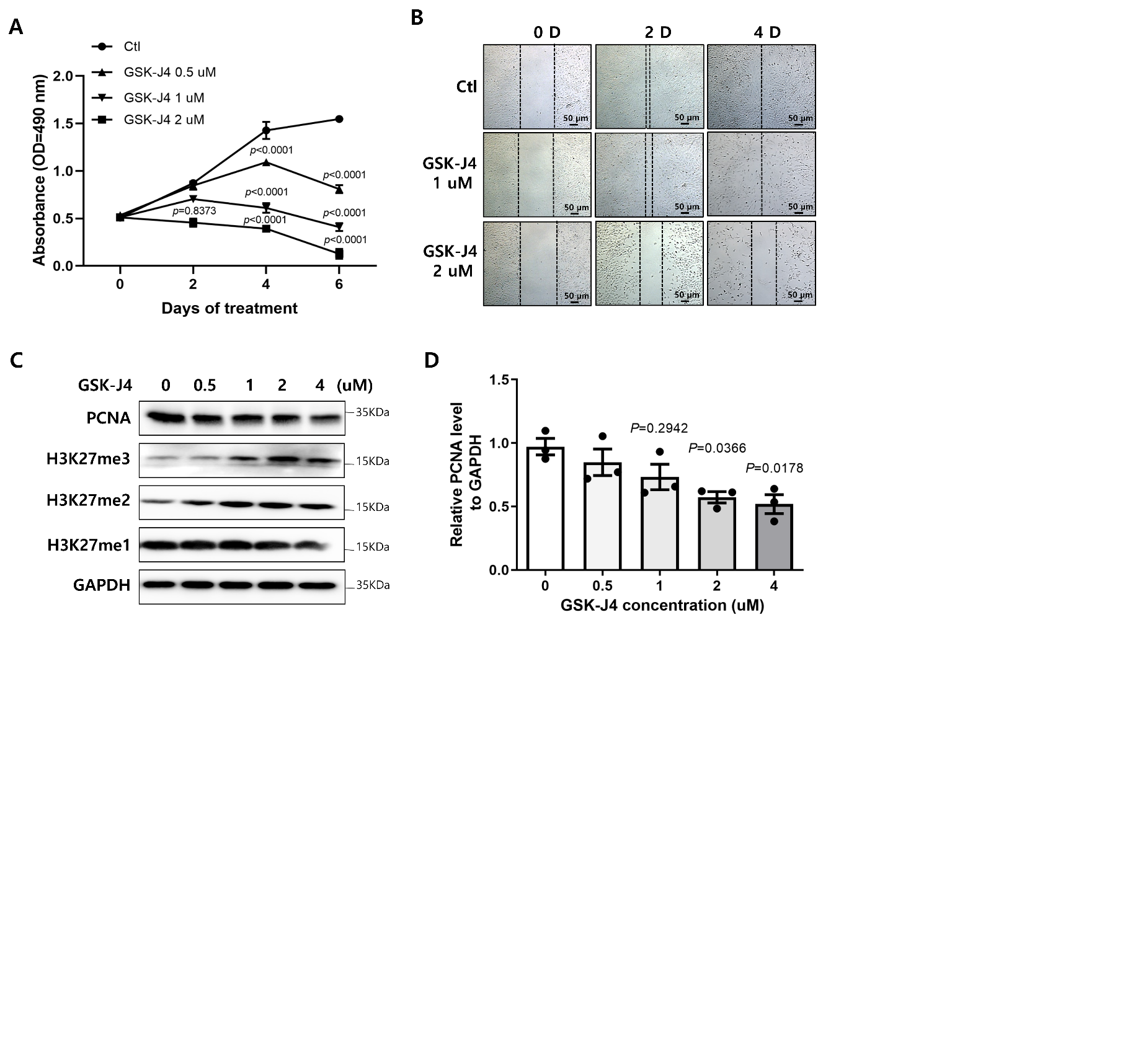


Figure S2. Effect of JMJD3 inhibition on EC function. A. ECs were cultured at 90 % confluence in a 96-well plate. Various concentration of inhibitor GSK-J4 (Selleckchem, S7070) or solvent were added. Primary EC proliferation was detected by MTS. Data were presented as mean ± SD for three replications. Significant differences between groups were analyzed by two-way ANOVA. B. GSK-J4 does-dependently suppressed EC migration *in vitro* wound-healing assay. ECs were cultured at 90 % confluence in a 12-well plate. A scratch wound was created physically using a sterile pipette tip. The cells were washed with phosphate-buffered saline to remove the debris and further cultured in EC medium. After addition of GSK-J4 or solvent (Ctl), the healing of the scratch wound was monitored at different time points by phase-contrast microscopy and photographed. Photos are representative of 3 independent experiments. C. GSK-J4 dose-dependently down-regulated the expression of PCNA in primary EC culture. ECs were cultured at 90 % confluence in a 12-well plate. Western blot was used to detect the expression of PCNA and methylation of H3K27 after treatment with GSK-J4 or solvent (Ctl) for 48 hr. D. Quantification of Western blot analysis (C) were shown. Data were presented as mean ± SD (n = 3). Significant differences between groups were analyzed by one-way ANOVA.
